# Occupational Exposure to Aerosolized Brevetoxins during Florida Red Tide Events: Effects on a Healthy Worker Population

**DOI:** 10.1289/ehp.7502

**Published:** 2005-02-10

**Authors:** Lorraine C. Backer, Barbara Kirkpatrick, Lora E. Fleming, Yung Sung Cheng, Richard Pierce, Judy A. Bean, Richard Clark, David Johnson, Adam Wanner, Robert Tamer, Yue Zhou, Daniel G. Baden

**Affiliations:** ^1^National Center for Environmental Health, Centers for Disease Control and Prevention, Atlanta, Georgia, USA;; ^2^Mote Marine Laboratory, Sarasota, Florida, USA;; ^3^National Institute of Environmental Health Sciences Marine and Freshwater Biomedical Sciences Center, University of Miami School of Medicine, Miami, Florida, USA;; ^4^University of Miami School of Medicine, Miami, Florida, USA;; ^5^Inhalation Toxicology Laboratory, Lovelace Respiratory Research Institute, Albuquerque, New Mexico, USA;; ^6^Children’s Hospital Medical Center, Cincinnati, Ohio, USA;; ^7^Florida Department of Health, Tallahassee, Florida, USA;; ^8^Center for Marine Science Research, University of North Carolina at Wilmington, Wilmington, North Carolina, USA

**Keywords:** aerosol, brevetoxins, *Karenia brevis*, lifeguards, pulmonary function, red tide, spirometry

## Abstract

*Karenia brevis* (formerly *Gymnodinium breve*) is a marine dinoflagellate responsible for red tides that form in the Gulf of Mexico. *K. brevis* produces brevetoxins, the potent toxins that cause neurotoxic shellfish poisoning. There is also limited information describing human health effects from environmental exposures to brevetoxins. Our objective was to examine the impact of inhaling aerosolized brevetoxins during red tide events on self-reported symptoms and pulmonary function. We recruited a group of 28 healthy lifeguards who are occupationally exposed to red tide toxins during their daily work-related activities. They performed spirometry tests and reported symptoms before and after their 8-hr shifts during a time when there was no red tide (unexposed period) and again when there was a red tide (exposed period). We also examined how mild exercise affected the reported symptoms and spirometry tests during unexposed and exposed periods with a subgroup of the same lifeguards. Environmental sampling (*K. brevis* cell concentrations in seawater and brevetoxin concentrations in seawater and air) was used to confirm unexposed/exposed status. Compared with unexposed periods, the group of lifeguards reported more upper respiratory symptoms during the exposed periods. We did not observe any impact of exposure to aerosolized brevetoxins, with or without mild exercise, on pulmonary function.

*Karenia brevis* (formerly *Gymnodinium breve*) is a marine dinoflagellate responsible for red tides that form annually in the Gulf of Mexico. *K. brevis* produces brevetoxins, the potent toxins that cause neurotoxic shellfish poisoning. The human health effects from consuming shellfish with high concentrations of brevetoxins in their tissues have been well documented. However, there is very little information describing human health effects from environmental exposures. In 1948 Woodcock stated, “It is ironic that we know the least about the aspects of the Florida red tide problem that poses the greatest public health hazard in terms of number of people affected” (Hemmert 1974). In 1999 Kirkpatrick et al. (2001) conducted a pilot study of the impact of environmental exposures to Florida red tide during a red tide research cruise. Although the number of participants was small, two scientists (both < 30 years of age and with no reported underlying pulmonary disease) reported difficulty in obtaining a deep breath and had decreases in pulmonary function parameters on a day when *K. brevis* cell counts were measured at > 8 million cells/L and the wind speed was higher than on other cruise days. In addition to the reports of effects on healthy individuals, there is evidence that laboratory sheep with induced asthma and people with asthma are adversely affected from exposure to aerosolized brevetoxins (Abraham et al. 2005; Fleming et al. 2005).

A pilot study of recreational beachgoers (Backer et al. 2003) found significant increases in reported upper and lower respiratory symptoms but no significant differences in spirometry test parameters during the exposed periods (when there was a red tide) when compared with symptom and spirometry data collected during an unexposed period (when there was no red tide). However, a number of limitations were associated with the study; for example, the study participants were a convenience sample of people who came to the beach and included some individuals with underlying respiratory illnesses (i.e., chronic obstructive pulmonary disease and/or a history of smoking), and many participants reported that they had been exposed to airborne red tide toxins for up to a week before the study and believed their symptoms had decreased during that time.

To begin to address the limitations of earlier studies, we wanted to identify a group of healthy individuals who were occupationally exposed to aerosolized brevetoxins during red tide events. We identified a population of full-time lifeguards working along Florida’s gulf coast who were willing to participate in a study. This group was interested in the health effects from inhaling aerosolized brevetoxins because the beaches in these communities do not close during onshore red tides and the lifeguards are required to conduct their normal activities, including staying in the beach guard towers for approximately 6 hr during each shift.

In addition to their potential exposures to aerosolized brevetoxins while conducting their work activities, lifeguards engage in some form of vigorous exercise (e.g., running, swimming) each workday. Investigators have reported that strenuous exercise (exercise that causes mouth breathing) can induce reversible bronchospasm in asthmatic individuals (Kirkpatrick et al. 1982). Because brevetoxin also causes bronchospasm in laboratory animal studies (Wells et al. 1984), it is possible that exercising on the beach during a time when there is red tide aerosol blowing onshore is a risk factor for developing respiratory symptoms or changes in pulmonary function.

Our objective was to conduct an occupational epidemiologic study in healthy workers to evaluate the reported symptoms and respiratory effects (using spirometry) from exposure to aerosolized red tide toxins and conduct a pilot study to assess whether mild outdoor exercise during a red tide event affects pulmonary function test (PFT) results or the number of self-reported symptoms.

## Materials and Methods

### Study protocol.

The study protocol was approved by the institutional review boards of the Centers for Disease Control and Prevention (Atlanta, Georgia); the Florida Department of Health (Tallahassee, Florida); and the University of Miami (Miami, Florida).

### Study population.

To be included in our study, an individual was required to be a full-time lifeguard working at one of the beaches in Sarasota or Manatee counties in Florida and at least 18 years of age. We recruited 28 full-time lifeguards who met our criteria and volunteered to be in the study. In general, these lifeguards are physically fit, participate in daily aerobic and weight training, and have little pulmonary disease.

### Pulmonary function tests.

Spirometry tests were done using portable 8-L dry rolling–seal volume spirometers (OMI, Houston, TX) by personnel trained using the course developed by the National Institute for Occupational Safety and Health (NIOSH 1997). The spirometry values of interest were forced vital capacity (FVC), forced expiratory volume in 1 sec (FEV_1_), FEV_1_/FVC percentage, forced expiratory flow between 25 and 75% of total FVC (FEF_25_–75%), and peak expiratory flow (PEF). We used the reference values from OMIS98 Spirometry software (version 3.18.7; OMI, Houston, TX) and the interpretation recommendations from the American Thoracic Society (1991) to compute predicted spirometric values. All study participants had at least three reproducible spirograms before and after visiting the beach, and the best values from these three spirograms were used for data analysis (American Thoracic Society 1991). The data were considered adequate if they conformed to standard guidelines for the collection and interpretation of spirometry measurements (American Thoracic Society 1995).

### Symptoms and respiratory effects study.

The lifeguards were interviewed using a questionnaire comprising questions about demographics and pulmonary health history. During a time when there was no red tide, we conducted pre- and postshift baseline PFTs and symptom surveys. The symptom survey included questions about upper respiratory symptoms (i.e., eye and throat irritation, nasal congestion, cough) and lower respiratory symptoms (i.e., chest tightness, wheezing, shortness of breath). The duration of the shift was 8 hr and included approximately 6 hr of exposure to marine aerosols. The pre- and postshift PFTs and symptom surveys were repeated during a time when there was a red tide.

### Exercise pilot study.

Most of the lifeguards regularly run on the beach as part of their physical training. However, running as an activity to increase minute ventilation and mouth breathing was not appropriate for this study because the concentrations of brevetoxins in the air are not consistent along the shoreline. Instead, we used a Monark Ergomedic weight ergometer (model 874E; Wynne International, New Dundee, Ontario, Canada). The ergometers were placed in the surf zone adjacent to a lifeguard tower and near one of the high-volume air samplers used to monitor brevetoxin concentrations. During a time when there was no red tide, a subgroup of the lifeguards performed two sets of spirometry tests (5 min apart), rode the ergometer for 5 min at a constant workload (90 cal/watt), and then performed another set of two spirometry tests (immediately after exercise and 15 min after exercise) and reported their symptoms. During the exposed period, the same subgroup of lifeguards repeated the study activities except that, because the results from the two sets of spriometry tests done immediately after exercise and 15 min after exercise were similar, the lifeguards performed only one set of spirometry tests before and after exercising. In addition, during the exposed period, the study activities were performed before and after their shift to assess any changes in morning and afternoon environmental conditions.

### Environmental monitoring.

During both studies, water samples were collected daily in 1-L glass bottles at 0830 hr, 1200 hr, and 1600 hr, from the surf zone adjacent to the study high-volume air sampler locations. A 20-mL subsample was taken from each bottle and fixed with Utermohl’s solution to provide *K. brevis* cell counts. The remaining water sample was transported to Mote Marine Laboratory and processed for liquid chromatography–mass spectrometry (LC-MS) analysis according to the procedure of Pierce et al. (2003).

In the laboratory brevetoxins were extracted by passing the water through a C-18 solid-phase extraction disk under vacuum (Ansys Technologies, Inc., Lake Forest, CA). The C-18 disks were then rinsed with reverse osmosis water to remove any remaining salts and eluted with methanol (Pierce et al. 2003). Brevetoxin analyses were performed by LC-MS using a ThermoFinnigan AqA high-performance liquid chromatograph–MS (Thermo Electron Corp., Manchester, UK). Mass spectral detection was obtained using an AqA single-quad system scanned from 204–1,216 AMU with AqA Max 40 V electrospray. The column was Phenomenex Luna C-18 5Fm 250 × 2 mm (Phenomenex, Torrance, CA); the solvent gradient was 0.3% acetic acid/H_2_O with initial 50:50 acetyl-nitrile (ACN)/H_2_O to 95:5 ACN/H_2_O over 30 min. The limit of detection (LOD) of the analysis for brevetoxins in seawater was 0.03 μg/L.

Air samples used to assess lifeguard exposure to brevetoxins in the air were collected using two instruments: high-volume air samplers and personal breathing zone samplers. Six high-volume air samplers (model TE-5000; Tisch Environmental, Inc., Village of Cleves, OH) with a single-stage filter were used; three were placed near the surf zone (about 25 m) approximately 100 m apart, and a second row of three was located approximately 50 m from the first row to provide an assessment of aerosolized toxin concentrations over time and space along the beach. The high-volume air samplers were fitted with a 20.32 × 25.4 cm glass-fiber filter (EPM2000; Whatman, Maidstone, UK). Filter samples were collected separately for morning and afternoon time periods (0830–1200 hr and 1230–1600 hr).

The traditional approach to individual occupational exposure assessment would be to have the lifeguards wear the personal samplers. However, there was concern that the personal samplers would interfere with emergency response activities or be destroyed by immersion in seawater. Instead, personal exposure was measured by placing personal samplers (IOM inhalable dust sampler; SKC, Inc., Eighty Four, PA) connected to a battery-operated pump (model 224-PCXR4; SKC, Inc.) on the lifeguard towers near the lifeguards’ breathing zones. A 25-mm glass-fiber filter (type A/E; Pall Life Science, Ann Arbor, MI) was used as the collection substrate. The sampling flow rate was 2 L/min controlled by a rotameter in the sampling pump.

Brevetoxins from the environmental and personal air samplers were recovered from the glass-fiber filters by extraction for 12 hr in acetone using a Soxhlet apparatus (Pierce et al. 2003). The extract was then transferred to vials using methanol for LC-MS and enzyme-linked immunosorbent assay (ELISA) analysis. Brevetoxin recovery from glass-fiber filters was verified by the addition of standard amounts of polyether brevetoxins PbTx-2 and PbTx-3 to each of three filters that were subsequently processed for LC-MS analyses.

A portable, self-contained weather station was used near the air sampling locations to monitor the air temperature, relative humidity, and wind speed and direction (Complete Weather Station; Davis Instruments, Hayward, CA). The weather station was solar powered, and the data were downloaded into a notebook computer.

### Statistical analyses.

Descriptive and other statistical analyses were performed using SAS statistical software (version 8.03; SAS Institute Inc., Cary, NC). We used the paired *t*-test for continuous data (i.e., the PFT results) and McNemar’s test for categorical data (i.e., the symptom questionnaire data) (Kleinbaum et al. 1982). We compared the changes in spirometry results and self-reported symptoms that occurred over a shift on a day when there was a red tide with changes that occurred over a shift on a day when there was no red tide. We also examined the impact of exercise on spirometry tests and self-reported symptoms during a time when there was a red tide with the impact of exercise during a time when there was no red tide. Specifically, we examined the impact of exercise on spirometry tests and symptom surveys conducted before the shift and after the shift. Finally, we compared the preshift changes in test results and symptom reports with the postshift changes.

## Results

The coastal environmental conditions present during the symptoms and respiratory effects study and the exercise pilot study are presented in Cheng et al. (2005b) (symptoms and respiratory effect study) and Table 1 (exercise study). The ambient temperatures were within typical ranges for the area during the symptoms and respiratory effects study; however, the ambient air temperatures were unusually low during both the exposed and unexposed periods for the exercise study. During the exposed period of the exercise study, onshore winds blowing from 10 to 25 km/hr provided a greater opportunity for exposure to aerosolized brevetoxins.

The environmental monitoring data, including *K. brevis* cell counts and brevetoxin levels in seawater samples and air samples during the symptoms and respiratory effects study and exercise pilot study, are presented in Table 2 (see also Pierce et al., in press). We found that the concentrations of *K. brevis* cells generally correlated with the concentrations of brevetoxins in seawater samples but did not correlate with concentrations of brevetoxins in the air. For example, on Lido Beach in September 2001, there were > 9 million *K. brevis* cells per liter on both 8 September and 10 September. The corresponding concentrations of brevetoxins in seawater samples were approximately 18 and 14 μg/L, respectively. However, the corresponding concentrations of brevetoxins in the air samples were approximately 20 and 6 ng/m^3^, respectively.

We found that the amount of brevetoxin in the air varied not only by time but also by geographic area (i.e., the specific beach where the samples were taken). For example, because there had been no reports of respiratory irritation or fish kills, we originally considered the May 2002 data collection period to be an unexposed period across all the beaches in our study. However, when the environmental sample analysis was completed for Nokomis Beach, the concentrations of *K. brevis* cells in seawater samples were low but above background levels (4,500 to > 36,000 cells/L), and there were measurable concentrations of brevetoxins in air samples on 2 of the 4 days on which data were collected. Thus, for this study, exposure status was determined separately for each individual lifeguard by day and by beach and was based on the air sample analyses.

There were 31 lifeguards eligible to be in the study; one declined to participate, and 30 were enrolled. We collected demographics and baseline spirometry data for 28 individuals (2 were lost to the study because they were called to military service). The demographics of the lifeguard study participants are presented in Table 3. Of the 28 lifeguards in our study population, 27 (96%) were white and 2 (7%) were female.

Baseline spirometry test results are presented in Table 4. As expected, the lifeguards are healthy with respect to lung function; that is, the PFT measurements were all at least 80% of the predicted values for the spirometry results based on reference values from OMIS98 Spirometry software, version 3.8.7, well above the minimum of 80% considered to be within the normal range. Also as expected, the measurements of lung function (FVC, FEV_1_, and PEF) were lower for female lifeguards than for male lifeguards.

On the basis of the environmental data for each beach on each day of our studies, we defined the unexposed period and two levels of exposed periods. The unexposed periods were days when there were no detectable levels of brevetoxins in air samples. The exposed days were defined as exposure level 1 (with detectable brevetoxin levels in air samples) and exposure level 2 (with brevetoxin levels > 10 ng/m^3^ in air samples). There were 17 lifeguards who worked a shift during a level 1 exposure and 13 who worked a shift during a level 2 exposure. There were 11 lifeguards who did not work a shift during an exposure period.

The results for self-reported symptoms for the symptom and respiratory effects study are presented in Table 5. Compared with the baseline data, there were significant increases in the reports of upper respiratory symptoms but not in the reports of lower respiratory symptoms during the periods of aerosolized brevetoxin exposure. In addition, there was a significant increase in self-reported headache in the exposure level 1 (any detectable brevetoxin in air samples) group.

The analyses of PFT results are presented in Table 6. We examined the changes in the individual test results during a shift (preshift data–postshift data). There were no significant changes in the PFT results during the unexposed period or during the exposure level 2 period. Compared with the unexposed period data, there were significant increases in FEV_1_ and PEF during the exposure level 1 period.

A subset of 11 lifeguards participated in the exercise pilot study to assess whether exercise during red tide events has an adverse impact on PFTs and/or the number of self-reported symptoms. When compared with the frequency of self-reported symptoms before exercising, there were no increases in the frequency of self-reported symptoms after exercising during the unexposed or the two exposed periods (data not shown).

During the unexposed period, and before their work shift, the lifeguards did two PFTs before exercising and two after exercising. The results from the two pre-exercise sessions were similar, and the results from the two postexercise sessions were similar (data not shown). There were no significant differences in PFT parameters when we compared the average pre-exercise results with the average postexercise results.

We also examined the changes in PFT values over the entire work shift (preshift and pre-exercise PFT results minus postshift and postexercise PFT values; data not shown). There were no significant changes in PFT parameter values during either the unexposed or the exposed periods.

## Discussion

In this study we examined the impact of occupational exposure to aerosolized red tide toxins on a group of full-time lifeguards. As part of their job activities, the lifeguards are required to be on the beach in guard towers, even if an onshore red tide is present. In addition, they are required to participate in a fitness maintenance program that includes running on the beach, swimming, and lifting weights. The purpose of our study was to examine the impacts of the lifeguards’ exposures to aerosolized brevetoxins during their normal shift and whether the impacts would be modified by exercise. The two end points used to measure the impacts were self-reported symptoms and spirometry tests.

During study periods when the potential for exposure to aerosolized brevetoxins was verified by environmental monitoring, the lifeguards in our study experienced symptoms consistent with longstanding and common anecdotal complaints of upper respiratory irritation made by residents and beach visitors during previous Florida red tides. These results are also consistent with symptom reports made by recreational beachgoers during red tide events involving similar levels of exposure (up to 36 ng brevetoxins/m^3^ of air).

Compared with nonexposure periods, the healthy lifeguards in our study reported more upper airway but not lower airway discomfort during the red tide exposure periods. There were statistically significant effects on some spirometry test parameters during exposure to red tide, but the changes were small and not clinically significant. In addition, we did not observe significant changes in any spirometry test parameters when we compared the effects of mild exercise during a nonexposure period with effects observed during an exposure period. These findings suggest that occupational exposures to low levels of aerosolized brevetoxins are not a serious health threat to this population. However, the upper respiratory irritation and discomfort caused by inhaling aerosolized red tide toxins can be substantial. Although these symptoms can be relieved by eliminating exposure, the lifeguards cannot leave the beach. To address this issue, we plan to examine the efficacy of different types particle face masks to determine which types may provide relief for the lifeguards and others who may not be able to avoid exposure to aerosolized brevetoxins during red tides.

Work by Cheng et al. (2005a) indicates that the size distribution of aerosols collected during red tides primarily reflects larger particles that are deposited in the upper respiratory tract. However, they also reported that a small but biologically significant fraction of the inhaled red tide aerosol was deposited in the lower airways. Perhaps, as concentrations of brevetoxins in the air increase, the amount of brevetoxin present in the smaller respirable particles also increases, thus increasing the effective dose of brevetoxins to the lower airways. This would be consistent with the our findings in an earlier study in which recreational beachgoers (Backer et al. 2003) reported experiencing increased lower respiratory irritation (wheeze, chest tightness, shortness of breath) when there were higher concentrations (up to 93 ng/m^3^), but not when there were lower concentrations, of brevetoxins in the air (Backer et al. 2003). In the present study, although the lifeguards were exposed for a much longer period of time (~ 6 hr) than the beachgoers were (average, 71 min; Backer et al. 2003), they were not exposed to high concentrations of brevetoxins and did not experience lower respiratory irritation.

We anticipated that when the lifeguards exercised they would increase their ventilation and effectively increase their dose of brevetoxin. However, they did not report any lower respiratory irritation after exercising during exposure to low levels of brevetoxins, again suggesting that an increased concentration of brevetoxin in the smaller, respirable particles when aerosolized brevetoxin concentrations are higher may be important in eliciting a lower airway response.

From a public health perspective, we would like to predict when aerosolized brevetoxins associated with Florida red tides will be at concentrations that can affect people on the beach. One possible way to quickly predict the presence of aerosolized brevetoxins would be to quantify the number of cells in seawater samples and extrapolate to airborne brevetoxin concentrations. However, we have found that cell concentrations do not correlate well with brevetoxin concentrations found in air samples collected during the same time period. We also found that brevetoxin concentrations in air samples varied considerably over a fairly small geographic area (e.g., among the beaches in our study) and were dependent on wind direction and speed as well as the presence of brevetoxins in the seawater itself. Unfortunately, the currently validated method to assess brevetoxins in air is gas chromatography–MS analysis, which requires considerable expertise and time to conduct. A user-friendly short-term test, such as a competitive ELISA (Naar et al. 2002), could be used to test routinely collected air samples and provide a database for public health officials responsible for public health on Florida’s gulf coast beaches.

There are a number of potential limitations associated with this study. We recruited healthy workers for our study, and thus the results cannot be generalized to all populations because they include groups that may be at increased risk because of underlying respiratory disease or other characteristics (Fleming et al. 2005). Another limitation is using self-reported symptom data, which can suffer from reporting bias. However, the actual exposure status of individual study participants was not known at the time the symptom data were collected but was established only after the air and water analyses had been completed, making it less likely that study participants could influence study results. For example, during the May 2002 data collection period, we assumed that all the lifeguards were unexposed. However, we found that those working at one beach (Nokomis) were actually exposed to substantial levels of aerosolized brevetoxins on some days.

Another study limitation could be the use of spirometry tests to assess the impact of exposure because we could not guarantee that study participants were providing their maximum effort during the tests. However, using the American Thoracic Society standards, it is almost impossible to reproduce three spirograms within the guidelines without maximal effort, making spirometry an objective measure of lung function.

## Conclusion

Anecdotal reports and some past studies have indicated that inhaling aerosolized brevetoxins associated with Florida red tides can cause respiratory irritation. This study has shown that when healthy lifeguards are occupationally exposed to low concentrations of brevetoxins in the air, they report upper airway irritation (i.e., eye irritation, nasal congestion, and cough) and headache. However, even when the lifeguards participated in mild exercise on the beach during a time when there were measurable levels of brevetoxins in the air, we did not detect changes in pulmonary function as measured using spirometry. Our results suggest that, for healthy people, exposure to low levels of brevetoxins in the air during Florida red tides is associated with temporary discomfort in the form of respiratory irritation but is not associated with acute adverse effects on pulmonary function. However, it would be appropriate to re-examine the health end points used in this study during periods of exposure to the higher levels of aerosolized brevetoxins (~ 100 ng/m^3^) that have been measured at Florida beaches.

## Figures and Tables

**Table 1:** Coastal environmental conditions during the data collection periods for the exercise study.

Date of exercise study	Temperature (°C)	Humidity (%)	Wind speed (km/hr)	Wind direction (% onshore)*^a^*
Unexposed period				
17 January 2003	12.2 ± 1.6	68 ± 5	25.6 ± 3.4	1
18 January 2003	8.3 ± 1.6	47 ± 5	10.9 ± 3.7	4
19 January 2003	13.3 ± 1.1	53 ± 7	12.4 ± 4.0	2
Exposed period				
29 March 2003	24.4 ± 0.5	83 ± 4	10.5 ± 5.4	58
30 March 2003	18.9 ± 2.2	84 ± 6	24.9 ± 6.0	44
31 March 2003	12.8 ± 1.1	32 ± 12	22.7 ± 2.6	0

Values are mean ± SD unless otherwise specified.

a Percentage of time the wind was blowing onshore. See Cheng et al. (2005a) for details about wind direction.

**Table 2:** *K. brevis* cell counts and PbTx concentrations in seawater and air samples.

Beach	Date	No. of *K. brevis* cells in seawater samples (cells/L)*^a^*	Brevetoxin levels in seawater samples (μg/L)*^b^*	Brevetoxin levels in air samples (ng/m^3^)*^c^*
Symptoms and respiratory effects study				
Siesta	3 May 2002	< LOD to 2,000	0.04 ± 0.4	1.11 ± 0.48
	4 May 2002	< LOD to 2,000	0.3 ± 0.4	1.16 ± 0.17
	5 May 2002	< LOD to 1,000	< LOD	< LOD
	6 May 2002	< LOD to 4,000	< LOD	0.05 ± 0.11
	7 May 2002	< LOD	< LOD	0.06 ± 0.14
	7 September 2001	< LOD to 1,000	27.9 ± 14.0	7.53 ± 3.86
	8 September 2001	< LOD	18.9 ± 8.0	9.94 ± 6.41
	9 September 2001	< LOD	8.6 ± 3.7	11.89 ± 7.07
	10 September 2001	388,500 ± 348,000	10.0 ± 3.3	2.40 ± 2.64
	11 September 2001	240,800 ± 223,800	12.3 ± 2.3	1.90 ± 1.66
Lido	3 May 2002	< LOD	< LOD	0.08 ± 0.17
	4 May 2002	< LOD	< LOD	0.08 ± 0.17
	5 May 2002	< LOD	< LOD	0.04 ± 0.09
	6 May 2002	< LOD	< LOD	< LOD
	7 May 2002	< LOD	< LOD	0.03 ± 0.06
	7 September 2001	12,100,000 ± 2,800,000	26.0 ± 16	26.90 ± 17.54
	8 September 2001	9,410,000 ± 278,000	18.3 ± 12.8	20.36 ± 27.16
	9 September 2001	799,000 ± 193,000	9.3 ± 6.6	17.43 ± 9.60
	10 September 2001	1,496,000 ± 663,700	13.8 ± 5.0	5.93 ± 7.26
	11 September 2001	79,399 ± 16,500	8.2 ± 2.4	1.32 ± 2.64
Nokomis*^d^*	3 May 2002	36,259 ± 22,100	2.1 ± 0.8	NA
	4 May 2002	18,000 ± 20,000	1.1 ± 0.7	6.4 ± 0.1
	5 May 2002	27,750 ± 11,200	0.6 ± 0.8	3.2 ± 2.0
	6 May 2002	29,500 ± 38,000	1.3 ± 1.3	< LOD
	7 May 2002	4,500 ± 3,100	0.1 ± 0.1	< LOD
	7 September 2001	NA	NA	NA
	8 September 2001	NA	NA	NA
	9 September 2001	382,500 ± 180,312	9.36 ± 8.25	49.21
	10 September 2001	608,500 ± 112,429	2.70	4.12
	11 September 2001	82,000 ± 9899	NA	17.58
Coquina*^e^*	3 May 2002	< LOD	< LOD	< LOD
	4 May 2002	< LOD to 1,000	< LOD	< LOD
	5 May 2002	< LOD	< LOD	< LOD
	6 May 2002	< LOD	< LOD	< LOD
	7 May 2002	< LOD	< LOD	< LOD
Exercise pilot study				
Unexposed period				
Siesta	17 January 2003	2,400 ± 1,400*^f^*	< LOD	< LOD
	18 January 2003	< LOD	< LOD	< LOD
	19 January 2003	< LOD	< LOD	< LOD
Exposed period				
Siesta	29 March 2003	180,600 ± 131,100	3.44 ± 1.93	36.57 ± 17.51
	30 March 2003	764,400 ± 263,700	14.01 ± 8.06	3.71 ± 2.63
	31 March 2003	96,300 ± 86,400	3.31 ± 3.74	< LOD

NA, not analyzed. Data are from the unexposed (May 2002) and exposed periods (September 2001) for the pulmonary function study and the unexposed (January 2003) and exposed periods (March 2003) for the exercise study. The values are mean ± SD of results from two seawater samples, of results from three high-volume samplers at Siesta and Lido beaches, and of results from personal sampler measurements at Nokomis and Coquina beaches. The values are presented by the specific beach where the measurements were made and by date.

a The LOD for *K. brevis* cells in seawater samples was 1,000 cells/L. The range of *K. brevis* cell concentrations is provided when 50% or more of the samples were < LOD. The mean ± SD is reported when cell concentrations were > LOD.

b The LOD for brevetoxins in seawater samples was 0.05 μg/L.

c The LOD for total brevetoxins in air samples was 0.05 ng/m^3^ for the high-volume samplers and 1.0 ng/m^3^ for the personal samplers.

d The air sampling results from Nokomis Beach are the averages ± SDs from two personal samplers used in May 2002 and the value for one personal sampler hung on the outside of the lifeguard tower in September 2001.

e The air sampling results from Coquina Beach are from one personal sampler hung on the lifeguard tower in May 2002. The LOD for total brevetoxins in air samples was 1.0 ng/m^3^ for the personal samplers. Coquina Beach did not have an onshore red tide during September 2001.

f Mean ± SD of samples with ≥1,000 cells/L; 30% of samples were < LOD.

**Table 3:** Demographics of the lifeguards who were enrolled in the study (*n* = 28).

Characteristic	No. (%)
Race	
White	27 (96)
Asian/Pacific Islander	1 (4)
African American	0
American Indian, Alaska native	0
Sex	
Female	2 (7)
Male	26 (93)
Mean age [years (range)]	35 (19–51)
Current smoker	0

**Table 4:** Baseline spirometry results for the lifeguards enrolled in the study (*n* = 26).

PFT parameter	Mean ± SD	Percent predicted ± SD*^a^*
Males only (*n* = 26)		
FVC (L)	5.71 ± 0.96	97.8 ± 17.1
FEV_1_ (L)	4.29 ± 0.73	92.9 ± 19.0
FEV_1_/FVC (%)	75.25 ± 6.35	94.1 ± 7.9
FEF_25–75%_ (L)	3.55 ± 0.99	
Peak flow (L/sec)	10.53 ± 1.86	
Females only (*n* = 2)		
FVC (L)	4.16 ± 0.37	147.2 ± 16.3
FEV_1_ (L)	3.65 ± 0.78	136.6 ± 0.1
FEV_1_/FVC (%)	87.86 ± 5.90	93.6 ± 9.0
FEF_25–75%_ (L)	4.12 ± 0.06	
Peak flow (L/sec)	8.36 ± 2.75	

For each lifeguard, we conducted spirometry tests in the morning before their shift, during a time when there was no red tide. For comparison, the estimated PFT values for a 180-pound adult male are FVC, 4.8 L; FEV_1_, 4.2 L; FEV_1_/FVC, > 70%; FEF_25–75%_, 4.5 L; peak flow, 9.5 L/sec (Scanlon et al. 1999).

a Percentage of predicted values as calculated by OMIS98 Spirometry software.

**Table 5:** Symptoms reported by study participants before and after going to the beach for the symptom and respiratory effects study.

	Exposure period		
Symptom	Unexposed (*n* = 27)	Exposure level 1 (*n* = 17)*^a^*	Exposure level 2 (*n* = 13)*^b^*
Upper respiratory			
Eye irritation	0	9 (52.9)*	7 (53.9)*
Nasal congestion	2 (8.7)	4 (23.5)*	3 (23.1)
Throat irritation	1 (4)	6 (35.3)	7 (53.8)*
Cough	1 (4)	9 (52.9)**	10 (76.9)**
Lower respiratory			
Chest tightness	0	1 (5.9)	1 (7.7)
Wheezing	0	0	1 (7.7)
Shortness of breath	0	2 (11.8)	0
Other symptoms			
Itchy skin	0	1 (5.9)	2 (15.4)
Headache	3 (12)	4 (23.5)*	1 (7.7)
Other	0	4 (23.5)	3 (23.1)
Screening symptom (not anticipated to be associated with aerosol exposure)			
Diarrhea	0	0	0

Values are number (%) of lifeguards who did not report the symptom before being on the beach but did report the symptom after being on the beach. The level of exposure was determined by the concentration of brevetoxins in the air.

a Detectable concentrations of brevetoxin (PbTx-2 plus PbTx-3) in air samples.

b Brevetoxin (PbTx-2 plus PbTx-3) concentrations > 10 ng/m^3^. Statistically significant using McNemar’s test:

* *p* < 0.05;

** *p* < 0.01.

**Table 6:** Changes in PFT results in study participants before and after their shifts.

	Exposure period		
PFT parameter	Unexposed (*n* = 28)	Exposure level 1 (*n* = 17)*^a^*	Exposure level 2 (*n* = 13)*^b^*
FVC^c^ (L)	0.08 ± 0.15	0.00 ± 0.13	−0.02 ± 0.17
FEV_1_^d^ (L)	0.07 ± 0.15	−0.03 ± 0.17*	0.03 ± 0.17
FEV_1_/FVC (%)	0.21 ± 3.41	−0.57 ± 2.05	0.63 ± 2.26
FEF_25–75%_^e^ (L)	0.03 ± 0.35	−0.08 ± 0.44	0.17 ± 0.38
Peak flow (L/sec)	0.24 ± 0.74	−0.21 ± 0.70*	−0.09 ± 0.69

Values are mean ± SD of the changes (preshift minus postshift) in the PFT parameters. The level of exposure was determined by the concentration of brevetoxins in the air.

a Detectable concentrations of brevetoxin (PbTx-2 plus PbTx-3) in air samples.

b Brevetoxin (PbTx-2 plus PbTx-3) concentrations > 10 ng/m^3^.

* Statistically significant from baseline values using a paired *t*-test: *p* < 0.05.
